# The Unfinished Synthesis?: Paleontology and Evolutionary Biology in the 20th Century

**DOI:** 10.1007/s10739-018-9537-8

**Published:** 2018-11-06

**Authors:** David Sepkoski

**Affiliations:** 1 Department of History, University of Illinois, Urbana-Champaign, Urbana, IL USA; 2 Max Planck Institute for the History of Science, Boltzmanstraße 22, 14195 Berlin, Germany

**Keywords:** Paleontology, Modern evolutionary synthesis, George Gaylord Simpson, Stephen Jay Gould, Punctuated equilibrium

## Abstract

In the received view of the history of the Modern Evolutionary Synthesis, paleontology was given a prominent role in evolutionary biology thanks to the significant influence of paleontologist George Gaylord Simpson on both the institutional and conceptual development of the Synthesis. Simpson's 1944 *Tempo and Mode in Evolution* is considered a classic of Synthesis-era biology, and Simpson often remarked on the influence of other major Synthesis figures – such as Ernst Mayr and Theodosius Dobzhansky – on his developing thought. Why, then, did paleontologists of the 1970s and 1980s – Stephen Jay Gould, Niles Eldredge, David M. Raup, Steven Stanley, and others – so frequently complain that paleontology remained marginalized within evolutionary biology? This essay considers three linked questions: first, were paleontologists genuinely welcomed into the Synthetic project during its initial stages? Second, was the initial promise of the role for paleontology realized during the decades between 1950 and 1980, when the Synthesis supposedly "hardened" to an "orthodoxy"? And third, did the period of organized dissent and opposition to this orthodoxy by paleontologists during the 1970s and 1980s bring about a long-delayed completion to the Modern Synthesis, or rather does it highlight the wider failure of any such unified Darwinian evolutionary consensus?

The received view among historians of science is that the three major “architects” of the Modern Evolutionary Synthesis in the 1940s were Ernst Mayr, Theodosius Dobzhansky, and George Gaylord Simpson. That is to say, the Synthesis was an endeavor inspired, respectively, by a systematist, a geneticist, and a paleontologist, and was a project devoted to uniting those three disciplines around common evolutionary questions. If this account is true, then this development represented a major boost for paleontology, which in the decades before the Synthesis occupied a decidedly marginal position with respect to evolutionary biology (Sepkoski 2012).

Indeed, many of the foundational works in the Synthesis era trumpeted the importance of paleontology for the project—after all, it was the paleontologists who provided much of the physical evidence that evolution had taken place in life’s past. For example, in the preface to his consciousness-raising *Evolution: The Modern Synthesis*, Julian Huxley wrote that “The time is ripe for a rapid advance in our understanding of evolution,” citing paleontology prominently among the several fields which had provided “new facts or new tools of research” (Huxley 1942, p. 8). The same year, in his *Systematics and the Origin of Species*, Mayr acknowledged past “misunderstandings” between paleontologists and biologists, urging a more unified approach in future evolutionary studies (Mayr 1942, pp. 291–292). And, in his own “official” contribution to the early Synthesis, *Tempo and Mode in Evolution* (1944), Simpson himself offered the view that despite previous mistrust “paleontologists and geneticists are learning tolerance for each other” fostered by the realization that “we do have problems in common and hope that difficulties encountered in each separate type of research may be resolved or alleviated by the discoveries of the other” (Simpson 1944, pp. xv–xvi).

This détente between paleontology and biology was highlighted by the leading role Simpson took in organizing the Synthesis: in 1942 (along with Dobzhansky) he worked to create the Committee on Common Problems in Genetics and Paleontology, and in 1944 (after returning from war service), he inherited its chairmanship, leading the group to recognition by the AAAS in 1946 as the re-named Society for the Study of Evolution, with the journal *Evolution* as its official organ (Cain 1993, p. 10). From its first issue, *Evolution* routinely featured papers by paleontologists like Simpson and his colleague at the American Museum of Natural History, the invertebrate paleontologist Norman Newell. Simpson, whose 1953 *Major Features of Evolution* (a heavily revised second edition of *Tempo and Mode*) enjoyed wide popularity and approval from neo-Darwinian biologists and paleontologists, continued to play a major role in public dissemination of the Synthesis project over the next decade, including contributing a lengthy essay on “Evolution and the History of Life” to the University of Chicago Centennial celebration of Darwin in 1959 (Tax ed. 1960). Two decades later, in his retrospective assessment of the era in *The Evolutionary Synthesis* (co-edited with William Provine), Mayr remarked that Simpson “was one of the most important architects of the synthesis” who “engineered the marriage of paleontology with genetics and more broadly with the rest of evolutionary biology” (Mayr and Provine 1980, p. 153).

From this brief sketch, it would seem that paleontology was indeed a major contributor to the Modern Synthesis. Simpson himself maintained this view throughout his life, even claiming in an unpublished autobiographical sketch that he had coined the term “Synthetic Theory of Evolution” (Simpson, “Remarks on Research and Publications,” 1). He frequently remarked that Dobzhansky’s *Genetics and the Origin of Species* (1937) had “most deeply influenced” his own theoretical understanding of evolution, and Simpson is widely credited with bringing an appreciation for modern population biology to paleontology.

There is just one problem with this account: if it is true, then why did so many members of the generation of paleontologists who followed Simpson—Stephen Jay Gould, Niles Eldredge, Steven Stanley, David Raup, Thomas Schopf, and others—sharply criticize the role allotted to paleontology by the rest of the evolutionary community in the decades following the publication of *Tempo and Mode*? Eldredge, for example, asserted that the consensus achieved by the Synthesis existed “only in the narrow sense.” Ultimately, he claims, what triumphed “was little more than hegemony achieved by a Fisherian population genetics view of the evolutionary process” that left little for paleontologists to contribute beyond mere documentation of history (Eldredge 1985, p. 29).^1^ Likewise, Gould—Eldredge’s co-author on the controversial theory of Punctuated Equilibria—agreed that while many of the initial synthetic works admitted a “pluralistic” vision of evolution, the 1940s saw a “hardening” of the Synthetic viewpoint, which he described as “neo-Darwinism and its insistence that cumulative natural selection leading to adaptation be granted pride of place as *the* mechanism of evolutionary change” (Gould 1983, p. 75). Like Eldredge, Gould concluded that this effectively pushed paleontology’s creative role in the Synthesis to the margins.

Gould’s claim about the hardening of the Modern Synthesis has received considerable scrutiny and criticism since it was advanced in the early 1980s, and it is not my intention to referee the subsequent debate. I will, however, ask three specific historical questions: first, were paleontologists genuinely welcomed into the Synthetic project during its initial stages? Second, was the initial promise of the role for paleontology realized during the decades between 1950 and 1980, when a number of prominent biologists and geneticists propelled a selectionist and adaptationist “neo-Darwinism” to textbook and disciplinary orthodoxy within evolutionary biology? And third, did the period of organized dissent and opposition to this orthodoxy by paleontologists during the 1970s and 1980s—which I have described elsewhere as a “paleobiological revolution”—bring about a long-delayed completion to the Modern Synthesis, or rather does it highlight the wider failure of any such unified Darwinian evolutionary consensus?

My answers to these questions are, in brief, yes, no, and perhaps. What this essay will show, however, is that these answers depend in large part on how particular terms and concepts are defined and who is employing them, and that their interpretations turn as much on institutional considerations (that is, questions about disciplinary orientation and status) as they do on intellectual or conceptual ones. The Modern Synthesis itself was, after all, as much a disciplinary engagement as it was a theoretical movement, and the question of paleontology’s role in any consensus achieved—a seat at the “high table” of evolutionary theory, as John Maynard Smith infamously put it—is part of a longer history of struggle for disciplinary autonomy and theoretical independence waged by paleontologists for more than a century.

## George Gaylord Simpson and the Modern Synthesis

If one were to ask a paleontologist—even one as outspoken as Gould or Eldredge—whether paleontology was better or worse off as a theoretical, evolutionary discipline after 1944, he or she would have little hesitation in asserting that it was better off, by a wide margin. Much of the credit for this is due to Simpson, whose *Tempo and Mode* genuinely did inspire a new way of approaching evolutionary questions in paleontology. And make no mistake: prior to the 1940s, paleontology was a decidedly second-class discipline in the eyes of many Darwinian biologists. Take, for example, the dismissive tone of Thomas Hunt Morgan, who remarked in 1916 that:

The geneticist says to the paleontologist, since you do not know, and from the nature of your case you can never know, whether your differences [in fossil series] are due to one change or to a thousand, you can not with certainty tell us anything about the hereditary units which have made the process of evolution possible. And without this knowledge there can be no understanding of the causes of evolution. (Morgan 1916, p. 27)

Likewise, despite reaching out an apparently friendly hand in *Evolution: The Modern Synthesis*, Huxley quickly withdrew it only a few pages later, writing that “paleontology is of such a nature that its data by themselves cannot throw any light on genetics or selection…. All that paleontology can do… is to assert that, as regards the type of organisms which it studies, the evolutionary methods suggested by geneticists and evolutionists shall not contradict its data” (Huxley 1942, p. 38).

Little wonder, then, that Simpson’s introduction to *Tempo and Mode* characterized past relations between paleontologists and biologists in such a negative light:

Not long ago paleontologists felt that a geneticist was a person who shut himself in a room, pulled down the shades, watched small flies disporting themselves in milk bottles, and thought he was studying nature…. On the other hand, the geneticists said that paleontology had no further contributions to make to biology, that its only point had been the completed demonstration of the truth of evolution, and that it was a subject too purely descriptive to merit the name “science.” (Simpson 1944, p. xv)

While Simpson’s characterization did not describe the attitude of *all* biologists (nor, indeed, did paleontologists of the preceding era entirely neglect evolutionary theory), the roots of this attitude can be traced all the way back to Darwin, who, in *The Origin of Species*, famously commented:

We have no right to expect to find in our geological formations, an infinite number of those fine transitional forms, which on my theory assuredly have connected all the past and present species of the same group into one long and branching chain of life…. [Rather] we ought only to look for a few links, some more closely, some more distantly related to each other; and these links, let them be ever so close, if found in different stages of the same formation, would, by most palæontologists, be ranked as distinct species. (Darwin 1859, pp. 301–302)

From the very beginning of modern evolutionary biology, then, deep suspicions existed about the ability for paleontology to make any contribution to evolutionary theory—suspicions that would not, in the end, be entirely overcome during the Synthesis.

This is not to say, however, that all paleontologists accepted this dim view of the role of paleontology or that they were content for their discipline to merely play “handmaid” to biology. In the decades prior to *Tempo and Mode,* a number of prominent paleontologists—from the Austrian Othenio Abel and the Germans Otto Schindewolf and Karl Beurlen to the Americans Henry Fairfield Osborn, William Diller Matthew, and B. F. Howell, among others—penned strong defenses of the role of paleontology in evolutionary studies (Sepkoski 2012, pp. 25–33). At stake was not only paleontology’s conceptual standing, but its institutional relationship with biology and geology as well: as Howell put it in his Presidential Address to the Paleontological Society in 1945, rather than looking “upon themselves as mere hand-maids to geology and to think of paleontology as nothing more than the tail on the geological dog,” paleontologists ought to consider their discipline as “a sister science to biology” and “an independent science, worthy of recognition as such” (Howell 1945, p. 375). Following Abel’s first introduction of the term, therefore, a number of paleontologists—including Simpson and eventually Newell—began referring to biologically-oriented, evolutionary paleontology as “paleobiology.”^2^

Though a mere 217 pages of text, Simpson’s *Tempo and Mode* set out to chart a new course for paleontology that would prove remarkably successful over the following decades. Simpson came to the project as a prominent vertebrate paleontologist steeped in the evolutionary tradition of Osborn and Matthew, his predecessors at the American Museum of Natural History; he was also, unlike most Anglophone paleontologists of his day, well-versed in the more theoretical German-language paleontology of Abel, Schindewolf, and others. But by his own account the crucial inspiration for his manifesto was his reading of Dobzhansky’s 1937 *Genetics and the Origin of Species*: as Simpson later put it, “The book profoundly changed my whole outlook and started me thinking more definitively along the lines of an explanatory (causal) synthesis and less exclusively along lines more nearly traditional in paleontology” (quoted in Mayr and Provine 1980, p. 456). Specifically, Simpson realized that the revolution underway in population genetics offered the possibility for paleontologists to re-imagine the fossil record in terms of the genetics of extinct populations, rather than merely as morphological series. In other words, Simpson asked how, given the morphological changes that could be observed in fossils, such changes could reveal patterns in the tempo (pace) and mode (mechanism) of evolution. Crucially, he believed that given its vastly longer timescale, paleontology could *uniquely* reveal causal features of evolution that biology or genetics could not perceive. This was as strong an argument as a paleontologist could make for his discipline’s prominent role in an emerging synthesis.

*Tempo and Mode* made a number of important theoretical contributions, including an elaboration of Sewell Wright’s notion of the “adaptive landscape,” which Simpson described as “selection landscapes” that could be correlated with geological time to reveal directional evolutionary trends. Here he argued—consistent with the viewpoints of Mayr and Dobzhansky—that Darwinian selective mechanisms could explain such trends, putting one of the final nails in the coffin of directional evolutionary theories like Lamarckism and orthogenesis. His perhaps most influential arguments had to do with the rate of evolution, which he argued could vary quite considerably in different lineages at different times. Using a technique known as survivorship analysis (borrowed from actuarial practices of life insurance companies), Simpson plotted curves estimating the longevity of particular taxonomic groups (say, bivalves versus land carnivores). These curves allowed him to estimate the mean evolutionary durations of particular taxa, revealing differences (bivalves, for example, tended to be around much longer than mammals) as well as commonalities in patterns of evolutionary development. In particular, he noted that general patterns of survivorship tended to follow the same diminishing parabolic curve: despite different overall longevities, higher taxa tended to decline steeply at first, and then to persist with a few remaining groups for a lengthy period of time.

This approach to survivorship analysis became a major feature of later evolutionary paleobiology, particularly in the hands of scientists like Leigh Van Valen (Simpson’s PhD student at Columbia) and David Raup. But of broader importance was the methodology Simpson employed, which had an explicitly quantitative, modeling orientation. Simpson believed that one of the major reasons for paleontology’s second-class status was its lack of mathematical sophistication; indeed, he had addressed this issue in an earlier textbook, *Quantitative Zoology*, which he co-authored with his wife Anne Roe, that attempted to teach basic statistical techniques to paleontologists and other students of natural history (Simpson and Roe 1939). In Simpson’s vision, paleontology would be a discipline that analyzed fossil *data* to discover evolutionary patterns, rather than merely producing descriptive morphological or taxonomic analyses of fossils themselves. This was very much in keeping with broader trends in “populational” biology, which had acquired great quantitative rigor through the work of R. A. Fisher, J. B. S. Haldane, Sewall Wright, and others in the first several decades of the 20th century (Provine 1971). It was also inspired by contemporary statistical systematics and character analysis promoted by German paleontologists, including Serge von Bubnoff and Rudolf Wedekind, whom Simpson cited appreciatively in *Quantitative Zoology* (Tamborini 2019). If Simpson can be credited with a single major achievement in synthesizing paleontology with genetics and population biology, it was in applying a measure of this quantitative rigor to paleontology so that members of the discipline could speak the same “language” as other evolutionary biologists. As Gould put it many years later, “This use of quantitative information provided Simpson’s second greatest departure from traditional paleontological practices…. Simpson introduced a novel style of quantification by drawing models (often by analogy) from demography and population genetics and applying them to large-scale patterns of diversity in the history of life” (Gould 1980a, pp. 158–159).

The importance of this approach for later studies in paleobiology during its “revolutionary” period in the 1970s and 1980s cannot be overstated: from the mid-1970s onwards, doing paleobiology meant studying patterns of diversification quantitatively. Simpson himself may not have achieved the striking results of his later followers (or even of his own colleagues like Newell), but *Tempo and Mode*—and the later *Major Features of Evolution*—was read by nearly every young paleobiologist between 1950 and 1980 and provided the crucial inspiration to attack big, evolutionary questions quantitatively. Later paleobiologists had the advantages of larger fossil databases to consult, more sophisticated (multivariate) statistical techniques, and, of course, digital computers. But Simpson was the unquestioned godfather of that later movement, and it is doubtful whether the revolution of the 1970s could have taken place without him.

This, however, does not answer the question of whether *in his day* Simpson achieved a genuine synthesis of paleontology with evolutionary biology—in fact, it fairly begs it. One way of addressing it is to examine whether Simpson’s own views about evolution fit comfortably within the Synthetic “orthodoxy” preached by Mayr and Dobzhansky. In many ways they did: Simpson consistently insisted that a populational approach should dominate paleontology and asserted that macroevolution—that is, long-term patterns of evolutionary development—could be explained by neo-Darwinian microevolutionary genetic mechanisms of selection and drift. In one crucial regard, though, *Tempo and Mode* was at odds with the view of Mayr and Dobzhansky, who basically carried on Darwin’s assumption that major breaks in the fossil record were the artifact of an imperfect preservation process. As Simpson put it, “The development of discontinuities between species and genera, and sometimes between still higher categories, so regularly follows one sort of pattern that it is only reasonable to infer that this is normal and that sequences missing from the record would tend to follow much the same pattern…. [T]he face of the fossil record really does suggest normal discontinuity at all levels” (Simpson 1944, pp. 98–99). Or, as he strikingly stated, “incompleteness is an essential datum and… can be studied with profit” (Simpson 1944, p. 105).

Based on the observation of these kinds of structural discontinuities in the fossil record, Simpson proposed that evolution proceeds with two distinct tempos: either slowly (bradytelic), which characterizes most lineages, or else more quickly (tachytelic), which appears in a significant minority of cases. To explain the latter—where evolution proceeds very quickly leaving few intermediate stages in the fossil record—Simpson introduced what Gould has called “his most striking and original contribution”: quantum evolution (Gould 1980a, p. 164). According to Simpson, cases of rapid evolution can be explained if we imagine a small, genetically-isolated population coming into disequilibrium, where it finds itself in an “inadaptive” portion of the selection landscape. Through random genetic mutations, selection, and drift, this population can be pulled to an adaptive peak (e.g., evolve) much more quickly than a larger population with greater geographic range and genetic diversity. He did not propose any accelerated genetic mechanism—such as Hermann J. Muller’s much-maligned genetic saltations—but merely pointed out that normal Darwinian processes might operate more quickly in some cases. Indeed, this idea was endorsed by Mayr himself in both *Systematics and the Origin of Species* and in a later landmark paper on allopatric speciation (Mayr 1954). However, while Simpson’s quantum evolution did not necessarily challenge Synthetic orthodoxy, his overall vision that it fit into was, as Gould put it, broadly pluralistic: “He wished to render macroevolution as the potential result of microevolutionary processes, not to rely dogmatically upon any single process. Although he favored selection-toward-adaptation as a primary (and dominating) theme, he explicitly denied that all evolution is adaptive and under selective control” (Gould 1980a, p. 165).

It is for this reason, Gould would argue, that Simpson had to be “disciplined” by Mayr and the other architects of the Synthesis during its period of “hardening” in the 1950s. The most notable evidence of this is the fact that, while quantum evolution was presented as the major conclusion of *Tempo and Mode*, it was virtually ignored in the revised 1953 *Major Features of Evolution*. Instead, Simpson now argued that “Phyletic splitting of lineages, including those from which higher categories up to the highest later develop, thus occurs by speciation at their bases…. The paleontological evidence cannot exclude the possibility of exceptions, but it confirms the conclusion in particular examples, and there is nothing in the record that requires or suggests exceptions” (Simpson 1953, p. 385). Quantum evolution, described in *Tempo and Mode* as “the dominant and most essential process in the origin of taxonomic units” (Simpson 1944, p. 206), was now characterized as merely “a limiting case on phyletic evolution” (Simpson 1953, p. 389).

Simpson left few clues in his notes and letters about what changed his mind, so it is difficult—if tempting—to speculate about the apparently abrupt about-face. Gould’s interpretation is that Simpson simply capitulated to pressure from Mayr, Dobzhansky, and others to avoid even the slightest suspicion of deviance from the selectionist orthodoxy of the Synthesis; while this hypothesis is plausible, there is no documentation that directly bears it out. It is undeniable, however, that Simpson’s choice significantly undercuts *Tempo and Mode*’s arguments for the disciplinary autonomy of paleontology within evolutionary biology. Quantum evolution had been the major example of the kind of original, theoretical result paleontology could contribute: a pattern of evolution not detectable by the methods of population biologists and geneticists. Implicitly, the stance of *Major Features* seems to indicate that paleontology should, after all, be content with documenting the gradual, phyletic patterns of evolution extrapolated from studies of living populations. I think it is ultimately unnecessary to speculate about Simpson’s own motivations for the change of heart, since it is undeniable that this was at least the interpretation the shift provoked for many later paleontologists. Indeed, several paleobiologists active during the revolutionary era reported reading *Tempo and Mode* only after having consumed *Major Features*, and being astounded by the radical nature of the earlier book’s arguments.^3^ In some cases (as with Gould and Eldredge), it was reading *Tempo and Mode* that inspired their own radical proposals—such as the theory of punctuated equilibria. Whatever Simpson’s intentions, then, the message that was communicated to later paleobiologists like Gould was that Simpson “unified paleontology with evolutionary theory, but at a high price indeed—at the price of admitting that no fundamental theory can arise from the study of major events and patterns in the history of life” (Gould 1980a, p. 170).

## Paleobiology After the Synthesis

In 1984, the geneticist John Maynard Smith published an essay in *Nature* entitled “Palaeontology at the High Table,” in which he generously welcomed paleontologists to the “high table” of evolutionary theory. This essay was remarkable for several reasons, not least of which because its author had previously been both a major proponent of the gene-selectionist viewpoint in evolutionary biology popularized by Richard Dawkins’ *The Selfish Gene* (1976) and a vocal critic of paleobiological theorizing by people like Gould. One should bear in mind that not only had Gould been championing punctuated equilibria and other recent—and unorthodox—contributions by paleontologists as challenges to received evolutionary theory for more than a decade, but he had only a few years earlier published his infamous essay “Is a New and General Theory of Evolution Emerging?” In that piece Gould opined that despite being “beguiled” by the “unifying power” of the Synthesis as a student during the 1960s, he had been “watching it slowly unravel as a universal description of evolution ever since,” concluding that “if Mayr’s characterization of the synthetic theory is accurate, then that theory, as a general proposition, is effectively dead, despite its persistence as textbook orthodoxy” (Gould 1980b, p. 120).

In fact, Maynard Smith’s *Nature* essay was a response to Gould’s 1984, Tanner Lectures at Claire Hall, Cambridge, where Gould offered some “Challenges to Neo-Darwinism and Their Meaning for a Revised View of Human CoAnsciousness” (Gould 1985). One might reasonably have expected to see the sharp-tongued Maynard Smith use his highly-visible platform to slap down the upstart Gould—as he had in the past and would again do in the future. But on this occasion Maynard Smith showed a curious restraint: rather than take exception to Gould’s complaints that paleontologists had been excluded from the Synthesis (many of which, quoted above, had been published in Gould’s 1980 chapter in *The Evolutionary Synthesis*), Maynard Smith essentially agreed. “It might be supposed,” Maynard Smith began, “that the contribution [of paleontology to evolutionary theory] would be crucial, but, at least until recently, that has not been so.” As he explained,

The palaeontologist G.G. Simpson was one of the main architects of the ‘modern synthesis’ that emerged in the 1940s, but his role was to show that the facts of palaeontology were consistent with the mechanisms of natural selection and geographical speciation proposed by the neonatologists (a term used by palaeontologists to describe the rest of us), rather than to propose novel mechanisms of his own. Since that time, the attitude of population geneticists to any palaeontologist rash enough to offer a contribution to evolutionary theory has been to tell him to go away and find another fossil, and not to bother the grownups. (Maynard Smith 1984, p. 401)

However, as Maynard Smith continued, “In the last ten years… this situation has been changed by the work of a group of palaeontologists, of whom Gould has been a leading figure.” He went on to cite punctuated equilibria, macroevolutionary hierarchy and species selection, and, particular, the study of mass extinctions as having had an important role in leading biologists to re-think—if not revise—some of evolutionary biology’s most cherished assumptions, leading him to the now famous invocation that “The palaeontologists have too long been missing from the high table. Welcome back” (Maynard Smith 1984, p. 402).

Maynard Smith’s statements could be—and have been—read in a variety of ways: as proof that paleontology had finally reached the promised land, as an attempted apology for past misdeeds and a tentative overture to improved future relations, or as further evidence of elitist paternalism on the part of geneticists (the “high table” is one of those distinctively British traditions in which the dining halls at Oxbridge colleges are segregated between the students, who sit together on the main floor, and the fellows, who eat at a raised table at the front of the room). In a sense, I detect elements of all three sentiments in his piece. Maynard Smith’s enthusiasm for some developments in recent paleontology—especially mass extinction studies, which he cited as having “the greatest impact… on the way we see the mechanisms of evolution”—was clearly genuine and was shared by many biologists at the time. There is also a note of subtle condescension in the essay, in which praise is dispensed ambivalently (he reported having “no problem” with a hierarchical theory of macroevolution, so long as it follows a number of fairly rigid neo-Darwinian conventions), and the tone is decidedly one of a senior colleague addressing a junior one. But on one issue Maynard Smith seems to have been in complete agreement with Gould: that the Modern Synthesis, as a genuine synthesis of paleontology and genetics, was a failure.

There are times when a historian should question his or her sources, and times when he or she should simply step back and let those sources have their say—and this is one of those times. Whether or not paleontology has since completed a delayed synthesis with evolutionary biology—and I suspect there are as many different opinions on that topic as there are paleontologists and evolutionary biologists—it seems fairly clear both from the historical record and from the mouths of the actors involved that whatever promise might have been extended in the 1940s was not achieved, at least until the mid-1980s or so. Mayr, writing both as an activist biologist during the Synthesis era and as a partisan historian several decades later, had a clear interest in characterizing the Synthesis as having been pluralistic and open in order to impose and celebrate his own distinctive view of what that synthesis should entail. Likewise, Gould and his compatriots in the 1970s and 1980s had conceptual and disciplinary motivations for arguing the reverse. In other words, there is no “right answer” to this question, but rather different perspectives that were conditioned by distinct disciplinary and theoretical agendas.

In the first instance, it literally put bread on their tables: Eldredge and Gould have both frankly acknowledged that without punctuated equilibria they would have had rather quiet, undistinguished careers, rather than becoming (to different extents) prominent public intellectuals with many scientific and popular accolades. Without a foil, however, punctuated equilibria had no teeth: indeed, one of the many criticisms of the theory that evolution proceeds through lengthy periods of morphological stasis, infrequently “punctuated” by bursts of rapid speciation, is that it was either unoriginal (having been anticipated by Simpson, Mayr, or other previous thinkers) or uncontroversial (for the stated reason that it did not require any novel evolutionary mechanisms) or both (Cain 2009; Sepkoski 2009). In fact, Gould and Eldredge themselves had difficulty over the years deciding whether the theory was perfectly orthodox in a Darwinian sense or wildly radical: in his magnum opus *The Structure of Evolutionary Theory* (2002). Gould seems to try to have it both ways.^4^ What is clear, though, is that both authors got much more mileage out of the radical interpretation than the benign one, since it attracted more press, more attention from outside biology, and more momentum for aggressive disciplinary change from within. The same can be said for other paleobiological contributions of the era, including so-called species selection (the notion that group-level traits, such as reproductive mode, can be selected for via Darwinian mechanisms), hierarchy (the idea that selection operates on a hierarchy of levels from the gene to the higher taxon with different rules at each), and mass extinction theory (for example, the proposal that the dinosaurs—and perhaps many other groups—became extinct because of an extraterrestrial impact event) (Stanley 1975; Vrba and Gould 1986; Gould 1985; Raup and Sepkoski 1986; Alvarez et al. 1980).

In the second place, paleobiologists of the 1970s and 1980s were seeking to distinguish what was new or different about their approach in order to gain disciplinary traction *within* geology and paleontology. By arguing that the Synthesis had failed or been incomplete, they were able to argue that their own sub-discipline—paleobiology—was the long hoped-for resolution to its ultimate completion. But, importantly, they could also make the case that completion of the Synthesis could not be accomplished within existing disciplinary structures—that is to say, with paleontologists isolated in museum or university geology departments, cut off from access to colleagues and resources in biology (and, particularly by the late 1980s, in molecular genetics) (Rainger 1993; Sepkoski 2012, ch. 1). This was an appeal both to geologists, who often controlled the very few positions allocated to evolutionary paleontologists (which usually were filled by stratigraphers), and to colleagues and administrators who could promote interdisciplinary work. Beyond the academy, important sources of funding—the National Science Foundation, for example—had few existing rubrics that were appropriate for paleobiologists. During the early 1980s, in fact, most US federal funding for paleobiology came from NASA’s astrobiology initiatives rather than from more traditional sources like the NSF. This began to change over the next decade or so, as outspoken paleobiologists like Eldredge and Gould successfully drew public attention to paleontology by capitalizing on—and some would argue by exaggerating—the radical nature of paleontological interventions into evolutionary biology.^5^

## An Unfinished Synthesis?

In his final, *magnum opus*, the massive volume *The Structure of Evolutionary Theory*, Gould reiterated earlier claims that the history of the Modern Synthesis underwent a period of “restriction” and “hardening” following the publication of Dobzhansky’s *Genetics and the Origin of Species* and Mayr’s *Systematics and the Origin of Species*. In Gould’s view (shared by his frequent collaborator, Eldredge), the Synthesis began with a more pluralistic outlook: “The original synthesists wanted to render all of evolution by known genetic mechanisms; but they tended to agnosticism about relative frequencies among the legitimate phenomena, notably on the issue of drift (and other random phenomena) vs. selection” (Gould 2002, p. 505). The chief culprit in this hardening, according to Gould’s account, was Mayr, who went too far in his goal to purge earlier, non-Darwinian approaches such as orthogenesis and genetic saltations, ultimately campaigning against more pluralistic ideas promoted by Dobzhansky (in the first edition of *Genetics*), Wright (by downplaying the role of drift), and even himself (modifying his initial views about allopatric speciation and macroevolution). This meant, Gould argued, that paleontologists like Simpson were forced to drop more aggressive claims for the theoretical autonomy of paleontology, as evidenced by Simpson’s *volte face* concerning quantum evolution between *Tempo and Mode* and *Major Features*.

If, as Gould claimed, the initial synthesis was pluralistic enough to admit mechanisms like drift and quantum evolution that acknowledged patterns of evolutionary discontinuity, then it follows that the resulting “hardened” synthesis was not a true synthesis. Accepting that logic for the moment, we can then ask whether the revolution in paleobiology of the 1970s and 1980s “completed” that synthesis. As of 1985, Eldredge was unwilling to allow that such a completion had occurred. That year he published his book *Unfinished Synthesis*, in which he maintained that “the current version of the ‘modern synthesis’ remains so unmoved by the data of systematics, paleontology, and large-scale ecology that … we still have a theory of evolution that is not directly addressed to the actual events of the history of life” (Eldredge 1985, p. v). Like Gould, Eldredge has argued throughout the rest of his career that a completion of that synthesis will occur only when the evolutionary community embraces a hierarchical theory of macroevolution that recognizes different mechanisms and modes of evolution occurring on different levels of selection. Not surprisingly, for both Gould and Eldredge the centerpiece of such a hierarchical theory is their theory of punctuated equilibria and the associated phenomenon of “species selection” (or “sorting”).

This may well be the case, and it is not my intention to try to settle the matter here. However, there are other reasons to believe that the intervention of paleobiologists during the revolutionary period was not decisive. As Michael Benton—a paleontologist long associated with the paleobiology movement who has spent his entire career in Britain (giving him, perhaps, more distance from the mostly US-centered events of the 1980s and beyond)—has argued, the Paleobiological Revolution, although a genuinely important episode, did not “set out the grounds within which modern palaeobiology operates,” because it mostly ignored the field of phylogenetic analysis (Benton 2013, p. 3). As Benton quite justifiably claims, much of the important research in paleontology since the 1990s on patterns of evolutionary development has focused on “comparative phylogenetic methods”—e.g., constructing phylogenetic trees—capable of shedding light on a variety of questions central to evolutionary biology:

what was the ancestral trait in a clade, how one trait (e.g. body size) affects another, how particular traits affect evolutionary rates, relative rates of evolution of different subclades in comparison with each other, whether the rate of evolution has decreased or increased through time, whether two subclades are evolving towards different evolutionary optima, how different traits relate to the likelihood of extinction, how population size has changed through time, whether there has been gene flow between particular species, when a species moved between land masses and the timings of accelerations and decelerations in trait evolution across clades and with respect to events such as climate changes or mass extinctions. (Benton 2013, p. 3)

Benton suggests a variety of reasons why the leading figures of the Paleobiological Revolution would have been resistant to phylogenetic approaches, but the essential point is that paleobiology, at least as it was understood in the 1980s, was out of step with the major new advances in biology (a claim that is fair when leveled at Gould, who remained ambivalent towards phylogenetics until the end, but not Eldredge, who was an early adopter of cladistic approaches).

Indeed, the paleobiological community now generally regards phylogenetics as an essential component of the discipline. Whereas the classic textbook *Principles of Paleontology,* coauthored by Steven M. Stanley and David M. Raup (two of the leading figures of the Paleobiological Revolution), all but ignored phylogenetics in both editions (1971 and 1978), the completely rewritten 2007 third edition, coauthored by Michael Foote and Arnold I. Miller (two “second generation” paleobiologists who studied under Raup and Jack Sepkoski), has a lengthy section on constructing cladograms and inferring evolutionary relationships through statistical analysis (Raup and Stanley 1971; Raup and Stanley 1978; Foote and Miller 2007). Likewise, the authoritative compilation of paleobiological theory and method, *Palaeobiology: A Synthesis*, published in 1990 by the British Palaeontological Association with more than 100 original chapters by the leaders in the field, includes only two chapters on phylogeny: very basic introductions to “Cladistics” and “Evolutionary Systematics” (Briggs and Crowther 1990). However, the 2001 second edition (which contains mostly new chapters) features six chapters on “Reconstructing Phylogeny,” including three that discuss molecular phylogeny (Briggs and Crowther 2001).

In fact, some of the most exciting work in paleobiology in the past decade has involved the correlation of fossil evidence (i.e., using traditional morphological techniques) with molecular data. The use of so-called “molecular clocks” (which estimate the rate of evolution in extinct lineages based on assumptions drawn from the rate of mutation in living analogs) has greatly clarified our understanding of the origin and flourishing of multicellular life and, in particular, the puzzle of the so-called “Cambrian explosion,” in which most of the major animal phyla present today appear (from the fossil record) to have burst into existence over just a few million years some 500 million years ago. For example, phylogenetic estimates now suggest that, in fact, multicellular organisms probably evolved much earlier—perhaps 700 million years ago—and underwent a period of explosive diversification because of environmental conditions during the later Cambrian. One of the leading figures in this new approach has been the Smithsonian Institution paleobiologist Douglas Erwin (another second-generation paleobiologist whose position is notably in the “Department of Paleobiology”), who explains, in an important coauthored paper on the Cambrian question, just how important phylogentic and other techniques have been:

The fossil record is now supplemented with geochemical proxies of environmental change; a precise temporal framework allowing for correlation of rocks in different areas of the world and evaluation of rates of evolutionary and environmental change; an increasingly rigorous understanding of the phylogenetic relationships between various living and fossil metazoan clades and their dates of origin, based largely on molecular sequences; and growing knowledge of the evolution of developmental processes through comparative studies of living groups. Collectively, these records allow an understanding of the environmental potential, genetic and developmental possibility, and ecological opportunity that existed before and during the Cambrian. (Erwin et al. 2011, p. 1091)

A particularly important feature of this research has been the role of evolutionary developmental genetics (which Erwin et al. stress), which has provided a framework for explaining how relatively large morphological changes can occur in geologically short intervals through modifications to developmental gene regulatory networks.

It goes without saying that many of these approaches—particularly molecular genetics and evo-devo—were unknown (or only dimly perceived) by the proponents of the Paleobiological Revolution.^6^ They can hardly be faulted for failing to include them in their claims for a more unified synthesis between paleontology and genetics. This leaves open the question, though, of whether integration of molecular and phylogenetic techniques since the 1990s marks, at long last, the completion of paleontology’s synthesis with evolutionary biology. The problem with this interpretation—aside from the dubious historiographic goal of fixing labels on complex historical developments—is that for the most part, these techniques were not known to the framers of the Modern Synthesis either. This is not to say that our current understanding of evolutionary biology is not built on a foundation laid down by Dobzhansky, Mayr, Simpson, and others—Charles Darwin, for example—but rather to question whether it is meaningful at all to speak of the Modern Synthesis as a continuing phenomenon, given the seismic shifts that have taken place in biology since the 1950s.

Rather, I think the Modern Synthesis is best understood as an event, coinciding roughly with the publication of a series of seminal books and papers between the 1930s and the early 1950s as well as a series of important disciplinary activities (like the founding of the Society for the Study of Evolution). In that context, it is fair to conclude that paleontology was an important part of the synthetic vision, but that this vision has been significantly eclipsed by subsequent events: the rise of computers as tools for phylogenetic and fossil diversity analysis, the molecular revolution, the Paleobiological Revolution of the 1970s and 1980s, the emergence of evo–devo and other major developments in geology (plate tectonics), ecology (theoretical modeling), and other fields. These events are self-contained phenomena, conditioned on contingent interactions in local contexts; there is no ultimate “core” we should seek for historically or philosophically to “Darwinism” or “The Modern Synthesis,” nor their final “completion.” From this perspective, paleontology’s role in evolutionary biology has continually evolved—as have other branches of evolutionary studies—and will surely continue to do so in the future in ways we historians cannot possibly predict from present conditions.

## Footnotes

1 Indeed, in 1985 Eldredge published a book outlining his alternative view of macroevolutionary hierarchy with the title *Unfinished Synthesis* (Eldredge 1985).

2 Importantly, Simpson was one of only a handful of contemporary English-speaking paleontologists to actively read and engage with German-language paleontological and biological literature (including authors like Abel and Schindewolf), which was decidedly more evolutionary and “paleobiological” at the time than the Anglo-American version. While he found many points of disagreement with those authors, they were nonetheless an important source of inspiration for his own engagement with evolutionary questions (Sepkoski 2012; Tamborini 2019).

3 Eldredge and Gould, especially, described reading *Major Features of Evolution* before *Tempo and Mode* (see Sepkoski 2014).

4 For example, in *Structure* Gould complained about the “major and persisting misunderstanding of punctuated equilibrium among neontologists,” which was “the false assumption that if we are really saying something radical, we must be staking a claim for a novel mechanism of speciation, or for a different (read non-Darwinian) style of genetic change” (Gould 2002, pp. 778–779). This is true in a strict sense, but it downplays claims Gould made elsewhere in the book that punctuated equilibria is the essential foundation for “revising and expanding the Darwinian paradigm on all three supporting legs of its essential tripod. For the punctuational style of change—disfavored by Darwin, who recognized the necessary status of gradualism within the logic of his world view—now emerges as a primary consequence of repairs and reinforcements upon all legs of the tripod” (Gould 2002, p. 927).

5 One of the crucial dilemmas in the establishment of paleobiology between the 1940s and the 1980s was the disciplinary and intellectual relationship between paleontology and geology, on the one hand, and biology, on the other. For the most part, invertebrate paleontologists have historically been housed in departments of geology, leaving them intellectually isolated from biologists. For this reason, invertebrate paleontology had, by the middle of the 20th century, developed mostly as a geologically-oriented, stratigraphic discipline. A rallying cry for paleobiologists, then, from Simpson to Gould was that paleobiology represented a properly *biological* orientation to past life—sometimes to the consternation of more traditionally-minded invertebrate paleontologists (see Sepkoski 2012).

6 Gould’s first major book, *Ontogeny and Phylogeny* (1977), is sometimes regarded as one of the foundational texts in the emergence of the field of evolution and development, particularly for its treatment of the role of heterochrony in evolution. However, this was an observation that few of his paleobiological compatriots noticed.
